# American Text Book of Prosthetic Dentistry

**Published:** 1913-10-15

**Authors:** 


### BIBLIOGRAPHY.

AMERICAN TEXT BOOK OF PROSTHETIC DENTISTRY. A new and the fourth edition of this valuable work is just issued from the press of the former publishers Messrs. Lea and Febiger. While the new editions is in many respects a reprint of the former edition there is evidence of much editorial supervision and restatement throughout the volume. The first chapter is practically a reprint of the former edition. The second chapter on Metallurgy has been revised to the extent of eliminating some of the methods of obtaining workable metals from the crude ores and briefer consideration of the less important metals used in the Prosthetic art. The third chapter on Porcelain Teeth has been revised by Dr. Ellison Hillyer. Much more space is given to the hand carving of teeth which is so little practiced today than is necessary and also to the commercial manufacture of teeth which no dentist does, at the same time the newer forms of teeth are not given proper recognition. The sixth, seventh and eighth chapters have been wholly rewritten by Dr. A. D. Grittman, and while some different technical methods are introduced we are not sure that the methods of impression taking with plaster for instance are an improvement on Dr. Burchards methods. The tenth chapter has been revised to include a statement of the Gysi method of bite taking and mandible function, which makes the chapter stronger than in the former edition. Some revision and some new material make chapter twelve on selection and adjusting the teeth on the occlusal frames more interesting and satisfactory from the technical as well as the artistic viewpoints.

The most surprising change in the work is the dropping of the chapter on cast metal plates. The editor gives as his reason for this ommission the statement that the casting process so far as plates are concerned is not sufficiently developed to make a definite statement practicable. We can't help thinking this is a serious error. The casting of aluminum and low fusing alloys has been so greatly perfected in the last five years as to make the construction of cast plates with these materials not only practicable but more definite than by any other process, with the possible exception of a properly vulcanized plate. While it is probably true that the cast metal plate lacks in tensile strength and is more soluable in the oral secretions, it can be accurately adapted and is sufficiently durable to warrant its construction wherever indicated. The use of the casting methods for saddle and bar plates of gold we regard as one of the best developments of the prosthetic art, as it can be used to supplant the extensive bridge work so frequently used to the invariable detriment of the remaining teeth if not to the patients general health. We trust before another edition of the work is made that the author will consent to throw out the chapter on crown and bridge work and substitute a well considered chapter on cast metal plates, as we are confident the cast metal plate is to be used more generally in the future than ever before.

The book is gotten up in the usual good style of this publishing house, and as a product of the printers art there is nothing further to be desired.

